# Preparation of Superhydrophobic P-TiO_2_-SiO_2_/HDTMS Self-Cleaning Coatings with UV-Aging Resistance by Acid Precipitation Method

**DOI:** 10.3390/nano15141127

**Published:** 2025-07-20

**Authors:** Le Zhang, Ying Liu, Xuefeng Bai, Hao Ding, Xuan Wang, Daimei Chen, Yihe Zhang

**Affiliations:** 1 Engineering Research Center of Ministry of Education for Geological Carbon Storage and Low Carbon Utilization of Resources, Beijing Key Laboratory of Materials Utilization of Nonmetallic Minerals and Solid Wastes, National Laboratory of Mineral Materials, School of Materials Science and Technology, China University of Geosciences, Xueyuan Road, Haidian District, Beijing 100083, China; 2 Department of Mechanical, Materials and Manufacturing Engineering, The University of Nottingham, University Park, Nottingham NG7 2RD, UK; 3 Laboratory and Equipment Management Department, China University of Geosciences, Xueyuan Road, Haidian District, Beijing 100083, China

**Keywords:** primary product of titanium dioxide, amorphous SiO_2_, superhydrophobic, self-cleaning coating, UV-aging resistance

## Abstract

The superhydrophobic coatings for outdoor use need to be exposed to sunlight for a long time; therefore, their UV-aging resistances are crucial in practical applications. In this study, the primary product of titanium dioxide (P-TiO_2_) was used as the raw material. Nano-silica (SiO_2_) was coated onto the surface of P-TiO_2_ by the acid precipitation method to prepare P-TiO_2_-SiO_2_ composite particles. Then, they were modified and sprayed simply to obtain a superhydrophobic P-TiO_2_-SiO_2_/HDTMS coating. The results indicated that amorphous nano-SiO_2_ was coated on the P-TiO_2_ surface, forming a micro–nano binary structure, which was the essential structure to form superhydrophobic coatings. Additionally, the UV-aging property of P-TiO_2_ was significantly enhanced after being coated with SiO_2_. After continuous UV irradiation for 30 days, the color difference (ΔE*) and yellowing index (Δb*) values of the coating prepared with P-TiO_2_-SiO_2_ increased from 0 to 0.75 and 0.23, respectively. In contrast, the ΔE* and Δb* of the coating prepared with P-TiO_2_ increased from 0 to 1.68 and 0.74, respectively. It was clear that the yellowing degree of the P-TiO_2_-SiO_2_ coating was lower than that of P-TiO_2_, and its UV-aging resistance was significantly improved. After modification with HDTMS, the P-TiO_2_-SiO_2_ coating formed a superhydrophobic P-TiO_2_-SiO_2_/HDTMS coating. The water contact angle (WCA) and water slide angle (WSA) on the surface of the coating were 154.9° and 1.3°, respectively. Furthermore, the coating demonstrated excellent UV-aging resistance. After continuous UV irradiation for 45 days, the WCA on the coating surface remained above 150°. Under the same conditions, the WCAs of the P-TiO_2_/HDTMS coating decreased from more than 150° to 15.3°. This indicated that the retention of surface hydrophobicity of the P-TiO_2_-SiO_2_/HDTMS coating was longer than that of P-TiO_2_/HDTMS, and the P-TiO_2_-SiO_2_/HDTMS coating’s UV-aging resistance was greater. The superhydrophobic P-TiO_2_-SiO_2_/HDTMS self-cleaning coating reported in this study exhibited outstanding UV-aging resistance, and it had the potential for long-term outdoor use.

## 1. Introduction

The cleanliness and UV-aging resistance of building exterior walls are important and challenging issues [1,2]. In recent years, superhydrophobic self-cleaning coatings have become a research hotspot due to their unique surface properties [3,4,5]. These surfaces can achieve the self-cleaning goal by the washing of rainwater, reducing the manpower and material resources required to clean the exterior walls of buildings [6]. Therefore, it demonstrated enormous potential in practical applications. These coatings were inspired by the surface of lotus leaves [7,8]. The synergistic effect of micro–nano binary structures and low-surface-energy materials led to its superhydrophobicity [9,10]. Superhydrophobic surfaces showed important applications in many fields, such as anti-icing [11,12], anti-fogging [13,14], corrosion resistance [15,16,17], and oil–water separation [18,19]. Specifically, in the field of self-cleaning, it had important application prospects. Water can easily roll off the surface and take away contaminants. However, building exterior walls were consistently exposed to outdoor environments, where UV light from sunlight can degrade the organic components of the coatings [20]. This led to the yellowing and aging of the coating. Ultimately, the coating surface lost its superhydrophobic self-cleaning performance. Researchers have attempted to improve the UV-shielding properties of coatings. Chen et al. [21] prepared a superhydrophobic polydimethylsiloxane/halloysite nanotube@CeO_2_ coating by the in situ assembly approach. It could shield 93% of ultraviolet B (UVB) and 84% of ultraviolet A (UVA). Hu et al. [22] reported a cellulose nanofibril/halloysite nanotube–zinc oxide hybrid film with superhydrophobic property. It showed high UV-blocking efficiency in UVA (95.7%), UVB (98.7%), and UVC (99.8%). Sun et al. [23] prepared a glass with hydrophobicity, self-cleaning, and UV-shielding properties by coating a transparent styrene–ethylene–butylene–styrene triblock copolymer coating on the glass. It can shield 98% of UV light. However, they only measured the UV-shielding rate of the coating but not the change in surface WCAs of the coating after continuous UV irradiation, that is, the UV-aging resistance of the coating. Therefore, it is crucial to investigate and develop superhydrophobic self-cleaning coatings with UV-aging resistance.

TiO_2_ was considered the most excellent and effective white coating in the world due to its non-toxic, harmless, strong covering power and excellent whiteness and glossiness [24,25,26]. Untreated TiO_2_ was named the primary product of TiO_2_ (P-TiO_2_), which was an intermediate product in the production process of TiO_2_. It was an important inorganic pigment widely used in various fields such as coatings, papermaking, plastics, rubber, ink, etc. P-TiO_2_ had similar performance to TiO_2_. However, it was cheaper than TiO_2_. Therefore, it has become a research hotspot in recent years. Due to uneven particle sizes, the photocatalytic performance of P-TiO_2_ was weak. However, when it was exposed to UV light, it still exhibited certain photocatalytic activity. When it was used as a coating, the photocatalytic performance seriously affects the UV-aging resistance and service life of the coating. To solve this problem, researchers have developed various methods. Surface coating of TiO_2_ was considered an effective strategy. Surface coating methods were mainly divided into three categories: inorganic coatings [27,28], organic coatings [29], and composite modification [30,31]. Inorganic coatings had been widely studied and applied due to their advantages in green environmental protection, mild and controllable reaction process, simple preparation process, and low cost. Dong et al. [32]. used the liquid deposition method and aluminum sulfate octahydrate as the aluminum source to coat rutile TiO_2_ with aluminum oxide. The dispersion of the sample was significantly improved. However, the improvement in UV-aging resistance was not significant. Zhang et al. [33]. prepared zirconia-coated rutile TiO_2_ composite materials using the chemical liquid deposition method with rutile TiO_2_ and ZrOCl_2_ as raw materials. The properties of this composite material had been improved compared to rutile TiO_2_. However, it cannot improve the UV-aging resistance of TiO_2_. SiO_2_ has a high refractive index and excellent UV-shielding ability [34]. The reflection and scattering properties of UV light by rutile TiO_2_ can be effectively improved when it was coated with nano-SiO_2_. Meanwhile, UV radiation can be absorbed by the electronic structure of nano-SiO_2_, and it was converted into lower-energy thermal energy. This can reduce the penetration and damage of UV light to the material. In addition, the generation of active functional groups of P-TiO_2_ under UV irradiation can be inhibited by the SiO_2_ layer on the surface of TiO_2_. Therefore, its photocatalytic performance was decreased, and its UV-aging resistance was enhanced.

The UV light emitted by sunlight can be divided into three regions according to wavelength: UVA (400–320 nm), UVB (320–280 nm), and UVC (280–200 nm) [35,36,37]. Nano-SiO_2_ showed extremely strong UV-shielding ability, with a UVA shielding rate of 88%, a UVB shielding rate of 85%, and a UVC shielding rate of more than 70% [38,39]. Nano-SiO_2_ has a stable chemical structure. Si atoms are tightly bound to O atoms through covalent bonds. This covalent bond has a high bond energy and can withstand the energy impact of UV photons. Under UV irradiation, the chemical bonds inside nano-SiO_2_ cannot be easily broken [40,41]. In addition, the surface of nano-SiO_2_ particles can reflect UV light. Due to its size being comparable to the wavelength of UV light, light scattering occurs when UV light is irradiated onto the surface of nano-SiO_2_ particles. This scattering effect will change the direction of UV radiation propagation. This prevents UV light from effectively penetrating the interior of the material [42,43]. This reduces the destructive effect of UV radiation on the internal structure of the material. This greatly reduces the damage of UV light to the substrate material under the coating, and it improves the UV-aging resistance of materials.

In this study, sodium silicate was used as the silicon source, and P-TiO_2_-SiO_2_ composite particles were prepared by coating P-TiO_2_ with nano-SiO_2_ particles by the acid precipitation method. The P-TiO_2_ material had similar properties to TiO_2_, but it was cheaper. This approach not only enhanced the UV-aging resistance of P-TiO_2_ but also formed the micro–nano binary structure, which was the infrastructure for forming superhydrophobic surfaces. After modification and spray, a superhydrophobic P-TiO_2_-SiO_2_/HDTMS coating with excellent UV-aging resistance was obtained. The UV-aging resistance was necessary for coatings to be used outdoors for a long time, which was important for its practical application.

## 2. Experimental Methods

### 2.1. Materials and Reagents

P-TiO_2_ was made from rutile-type TiO_2_ (99.50%), and it was provided by Jiaozuo Changbaite Group Co., Ltd., Jiaozuo, China. Its oil absorption capacity was 17.52 g/100 g, and its average particle size was approximately 200 nm. Hexadecyltrimethoxysilane (HDTMS), with the molecular formula (CH_3_(CH_2_)_15_Si(OCH_3_)_3_, was supplied by Shanghai Macklin Biochemical Technology Co., Ltd., Shanghai, China. Analytical-grade sodium silicate, oxalic acid, and anhydrous ethanol were purchased from Tianjin Huasheng Chemical Reagent Co., Ltd., Tianjin, China. Analytical-grade NaOH was obtained from Beijing Chemical Works, Beijing, China. Epoxy resin was used as commercial AB adhesive.

### 2.2. Preparation of P-TiO_2_-SiO_2_ Composite Particles

In this study, P-TiO_2_-SiO_2_ composite materials were prepared by the acid precipitation method [44,45]. P-TiO_2_ was the raw material. Na_2_SiO_3_ was the pre-dispersant, and sodium silicate was the silicon source. pH was adjusted by a 10% NaOH solution and a 10% oxalic acid solution. The silicon source and pH regulator were added simultaneously. The process included coating, aging, washing, drying, and grinding.

The specific preparation steps are as follows:

1. A total of 20 g P-TiO_2_ was added to 100 mL of distilled water, and it was ultrasonically dispersed. Then, it was heated to 85 °C in a thermostat water bath.
2. The pH value was adjusted to 10 by a 10% NaOH solution, and 0.03 g of sodium silicate was added. Then, it was stirred and dispersed by a magnetic stirrer for 20 min.
3. A total of 8 g sodium silicate was added to 100 mL of water. Then, it was ultrasonically dispersed for 20 min. The prepared sodium silicate solution and 10% oxalic acid solution were added to a peristaltic pump. The silicon source was slowly dripped, and the pH of the suspension was maintained at 9.
4. After all the silicon sources were added dropwise, the suspension reacted for 90 min. Then, it was matured at 25 °C for 120 min.
5. The suspension was centrifuged and washed. Then, the precipitate was dried at 60 °C and grinded to obtained the P-TiO_2_-SiO_2_ composite material.

The reaction equation is as follows:

Na_2_SiO_3_ + H_2_C_2_O_4_→H_2_SiO_3_ + Na_2_C_2_O_4_ (1)

H_2_SiO_3_→SiO_2_ + H_2_O (2)

### 2.3. Preparation of the P-TiO_2_-SiO_2_/HDTMS Coating

**Figure 1:**
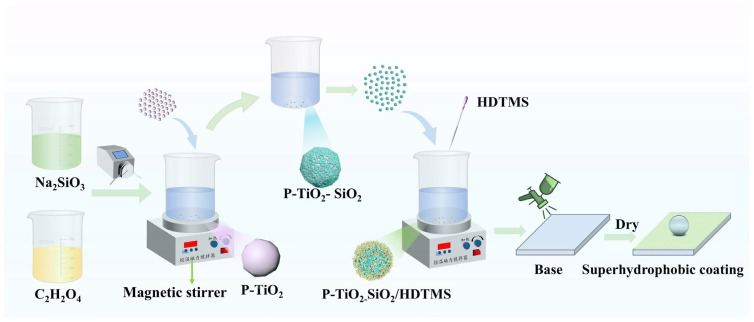
Schematic diagram of the preparation process of the superhydrophobic P-TiO_2_-SiO_2_/HDTMS coating.

The preparation process of the P-TiO_2_-SiO_2_/HDTMS coating is illustrated in Figure 1. First, 2 g of the P-TiO_2_-SiO_2_ composite particles was added to 10 g of ethanol. Then, it was stirred for 10 min. Subsequently, 0.4 g of HDTMS was added and stirred for 60 min to form the P-TiO_2_-SiO_2_/HDTMS slurry. This slurry was sprayed onto the substrate surface by a small sprayer, and the P-TiO_2_-SiO_2_/HDTMS coating was obtained after drying [46,47].

The preparation process of the P-TiO_2_-SiO_2_/HDTMS coating with epoxy resin was similar to that of the P-TiO_2_-SiO_2_/HDTMS coating. Epoxy resin and HDTMS were added simultaneously, and the quality ratio of epoxy resin and HDTMS was 1:10. The other preparation steps were the same.

### 2.4. Experimental Methods and Characterization

The wettability of the coating surface was characterized by WCAs and WSAs. Its UV-aging resistance was analyzed by calculating ΔE* and Δb*. The photocatalytic degradation performances of samples were also tested. The structures and reaction mechanisms of samples were characterized by different methods. Their details are shown in the Supplementary Materials.

## 3. Results and Discussion

### 3.1. Properties and Surface Structure of the P-TiO_2_-SiO_2_/HDTMS Coating

The P-TiO_2_-SiO_2_/HDTMS coating prepared in this study exhibited excellent superhydrophobic property and UV-aging resistance. Additionally, it demonstrated good adaptability to different droplets and substrates. The performance and surface structure of the coating were tested and characterized.

#### 3.1.1. Wettability and Adaptability of the P-TiO_2_-SiO_2_/HDTMS Coating Surface

**Figure 2:**
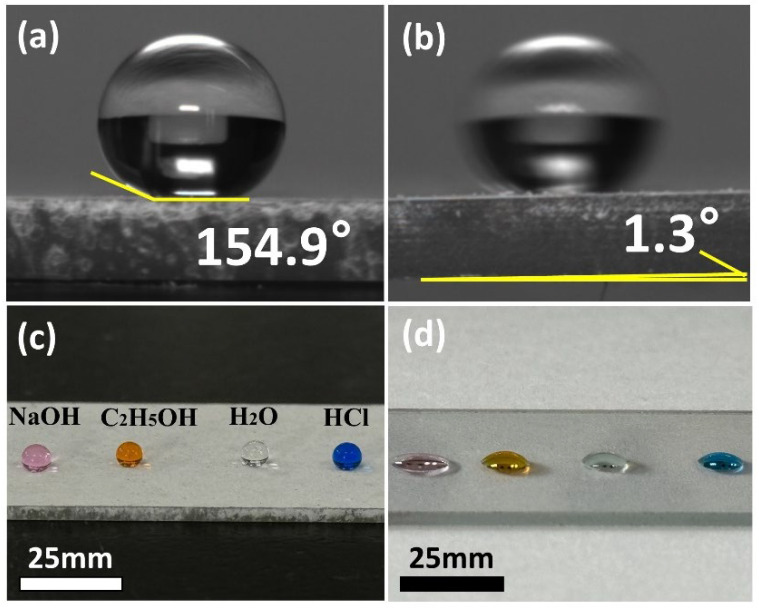
(**a**) WCA and (**b**) WAS on the P-TiO_2_-SiO_2_/HDTMS coating surface, and the wetting states of different droplets on the (**c**) P-TiO_2_-SiO_2_/HDTMS coating surface and (**d**) glass slide surface.

Figure 2a,b show the WCA and WSA on the P-TiO_2_-SiO_2_/HDTMS coating surface, respectively. In Figure 2a, a water droplet on the coating surface was nearly spherical with the WCA of 154.9° and the WSA of only 1.3°. Figure 2c,d illustrate the wetting behavior of liquids with different properties on the P-TiO_2_-SiO_2_/HDTMS coating and glass slide surfaces. As shown, the droplets with different properties formed nearly spherical shapes on the P-TiO_2_-SiO_2_/HDTMS coating surface. As a comparison, they almost completely spread on the glass slide surface. This indicated that the coating exhibited significant repellency to these liquid droplets.

**Figure 3:**
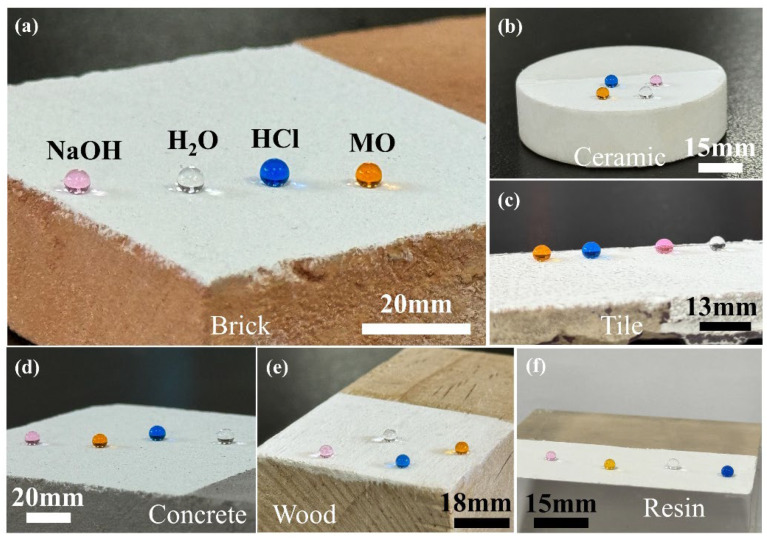
The wetting states of different droplets on the coating surface and the adaptability of the coating to different substrates: (**a**) brick, (**b**) ceramic, (**c**) tile, (**d**) concrete, (**e**) wood, (**f**) resin.

To evaluate the adaptability of the P-TiO_2_-SiO_2_/HDTMS coating to different substrates, the P-TiO_2_-SiO_2_/HDTMS slurry was sprayed onto the (a) brick, (b) ceramic, (c) tile, (d) concrete, (e) wood, and (f) resin surfaces to form coatings. Subsequently, NaOH droplets (pH = 11, dyed with Rhodamine B), water, HCl droplets (pH = 2, dyed with methylene blue), and methyl orange (MO) droplets were placed on the P-TiO_2_-SiO_2_/HDTMS coating surfaces, as shown in Figure 3. It can be observed that all these droplets formed nearly spherical shapes on the different substrates with WCAs greater than 150°, demonstrating superhydrophobic effects. This indicated that the P-TiO_2_-SiO_2_/HDTMS self-cleaning coating exhibited excellent adaptability to the aforementioned substrates with different properties.

#### 3.1.2. Self-Cleaning Property of the P-TiO_2_-SiO_2_/HDTMS Coating

**Figure 4:**
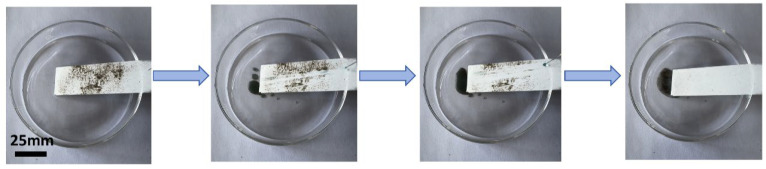
Self-cleaning performance of the P-TiO_2_-SiO_2_/HDTMS coating.

To evaluate the self-cleaning property of the P-TiO_2_-SiO_2_/HDTMS coating, it was placed in a glass dish at approximately 10° to the horizontal plane. Sand was sprinkled onto the coating surface. Subsequently, water (dyed with methylene blue) was dropped from the top of the coating by a syringe. Water droplets rolled off and carried away the sand particles from the surface when water droplets contacted the coating surface. Then, the self-cleaning goal was achieved. The process is shown in Figure 4.

#### 3.1.3. UV-Aging Resistance of the As-Prepared Coating

**Figure 5:**
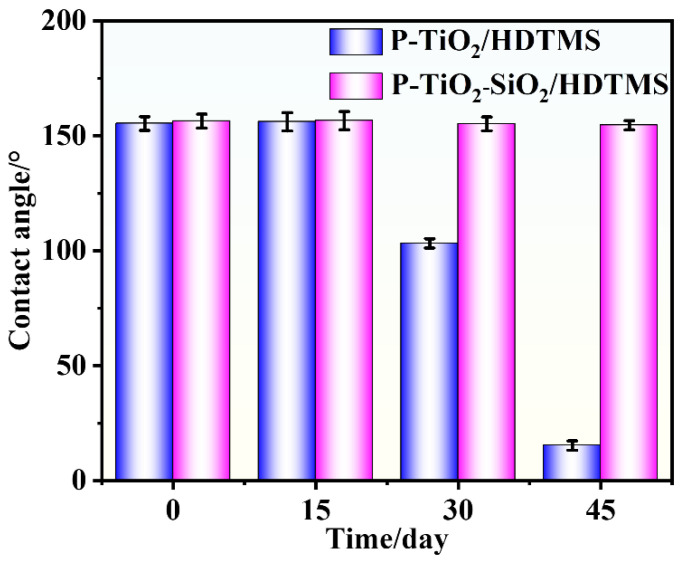
Changes in the WCAs of the P-TiO_2_-SiO_2_/HDTMS and P-TiO_2_/HDTMS coatings after UV irradiation for 45 days.

Figure 5 presents the result of the UV-aging resistance of the P-TiO_2_-SiO_2_/HDTMS coating. The WCAs and WSAs on the surface of the coating showed no significant changes after UV irradiation for 45 days. In contrast, the WCA of the P-TiO_2_/HDTMS coating decreased to 103.2° after UV irradiation for 30 days, losing its superhydrophobic property. After UV irradiation for 45 days, the WCA further decreased to 15.3°, and water droplets nearly completely spread on its surface. Furthermore, the UV-aging resistance of the P-TiO_2_-SiO_2_/HDTMS coating was significantly better than our previous research. The WCAs of the sericite–TiO_2_/HDTMS and sericite–rutile/HDTMS coatings decreased significantly after UV irradiation for 40 h [1] and 80 h [48]. These results demonstrated that SiO_2_ effectively enhanced the UV-aging resistance of the coating.

#### 3.1.4. Mechanical Strength of the As-Prepared Coating

**Figure 6:**
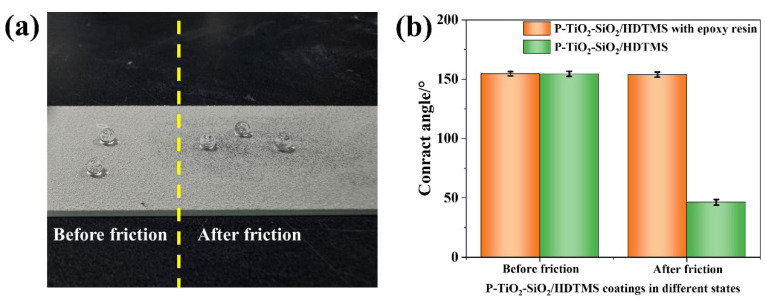
(**a**) The wetting states of water droplets on the P-TiO_2_-SiO_2_/HDTMS coating with epoxy resin before and after friction; (**b**) changes in the WCAs of P-TiO_2_-SiO_2_/HDTMS with epoxy resin and P-TiO_2_-SiO_2_/HDTMS coatings before and after friction.

The mechanical strength of the P-TiO_2_-SiO_2_/HDTMS coating can be improved by adding epoxy resin as a binder. A 360-grit sandpaper was placed on the surface of the coating, and a 50 g weight was pressed onto the sandpaper. The sandpaper was repeatedly pulled in the horizontal direction five times. Then, the wetting states of water droplets on the coating and the change in the WCA of the coating surface were measured, as shown in Figure 6. The results showed that the coating after friction also displayed superhydrophobic property, and the WCAs of the coating did not decrease obviously after friction. As a comparison, the WCAs of the coating surface without adding epoxy resin decreased to 46.3° after friction, and it lost superhydrophobicity. This indicated that the coating with epoxy resin had excellent mechanical strength.

#### 3.1.5. Roughness of the Surfaces

**Figure 7:**
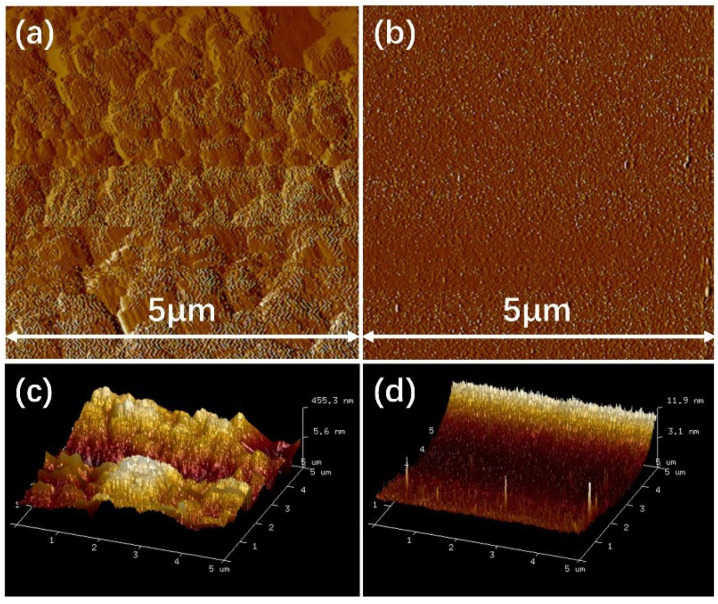
(**a**,**b**) Two-dimensional morphologies of the P-TiO_2_-SiO_2_/HDTMS coating and a glass slide surface; (**c**,**d**) 3D morphologies of the P-TiO_2_-SiO_2_/HDTMS coating and a glass slide surface.

Figure 7a,c display the 2D and 3D topographies of the P-TiO_2_-SiO_2_/HDTMS coating, while Figure 7b,d display the 2D and 3D topographies of the glass slide surface. It was clear that the glass slide surface was relatively smooth, with an average roughness of only 1.92 nm. In contrast, the P-TiO_2_-SiO_2_/HDTMS coating surface exhibited a hilly and uneven texture, with an average roughness of 192 nm. This illustrated that the P-TiO_2_-SiO_2_/HDTMS coating significantly increased the surface roughness of a glass. The rough surface structure was the foundational condition for a superhydrophobic surface.

#### 3.1.6. Surface Morphology and Thickness of the Coating

**Figure 8:**
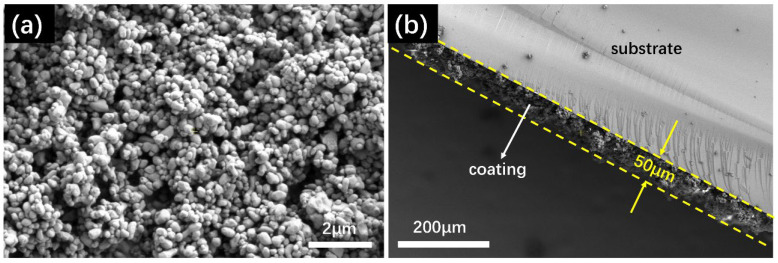
(**a**) Surface morphology of the coating; (**b**) the cross-sectional image of the coating.

Figure 8a shows the surface morphology of the P-TiO_2_-SiO_2_/HDTMS coating with epoxy resin. It can be seen that the stacking of P-TiO_2_-SiO_2_ particles forms a rough surface structure. Figure 8b shows the cross-sectional image of the P-TiO_2_-SiO_2_/HDTMS coating with epoxy resin. It can be seen that the average thickness of the coating was about 50 μm.

### 3.2. Mechanism for Improving the UV-Aging Resistance of the As-Prepared Coating

The UV-aging resistance of the P-TiO_2_-SiO_2_/HDTMS coating was attributed to the amorphous nano-SiO_2_ on the P-TiO_2_ surface, which reduced the photocatalytic activity of P-TiO_2_. The UV-aging resistance, photocatalytic degradation performance, and their mechanisms of action on P-TiO_2_-SiO_2_ were tested and discussed.

#### 3.2.1. UV-Aging Resistance of P-TiO_2_-SiO_2_ Composite Particles

**Figure 9:**
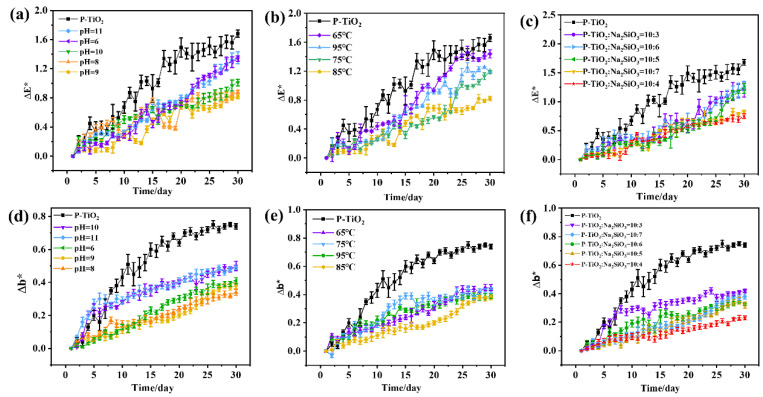
The ΔE* of the P-TiO_2_ and P-TiO_2_-SiO_2_ coatings prepared under different (**a**) pH values, (**b**) temperatures, and (**c**) dosages; the Δb* of the P-TiO_2_ and P-TiO_2_-SiO_2_ coatings prepared under different (**d**) pH values, (**e**) temperatures, and (**f**) dosages.

To investigate the influence of P-TiO_2_-SiO_2_ composite particles prepared under different conditions as fillers on the UV-aging resistance of coatings, they were placed in a UV accelerated aging machine. The ΔE* and Δb* of the coatings were measured, as shown in Figure 9. Figure 9a–c depict the ΔE* of coatings prepared with P-TiO_2_-SiO_2_ composite particles as fillers, which were prepared under different conditions. The results indicated that the ΔE* of coatings with P-TiO_2_-SiO_2_ composite particles as fillers were significantly lower than that of coatings with P-TiO_2_. When pH = 9, the temperature was 85 °C, and the mass ratio of P-TiO_2_ and Na_2_SiO_3_ was 10:4; the ΔE* of the coating prepared with P-TiO_2_-SiO_2_ as a filler only increased to 0.75 after UV irradiation for 30 days. Under the same conditions, the ΔE* of the coating with P-TiO_2_ as the filler increased to 1.68. It was obvious that the color change in the coating with P-TiO_2_-SiO_2_ as the filler was minimal. Figure 9d–f show the Δb* of coatings prepared with P-TiO_2_-SiO_2_ composite particles as fillers under different conditions. The results revealed that the Δb* of the coating with P-TiO_2_ as the filler increased to 0.74 after UV irradiation for 30 days, while the Δb* of the coating with P-TiO_2_-SiO_2_ as the filler increased only to 0.23 under the same conditions. This demonstrated that the coating with P-TiO_2_ as the filler turned extremely yellow. In summary, the SiO_2_ coating effectively enhanced the UV-aging resistance of P-TiO_2_.

#### 3.2.2. Photocatalytic Degradation Performance of P-TiO_2_-SiO_2_

**Figure 10:**
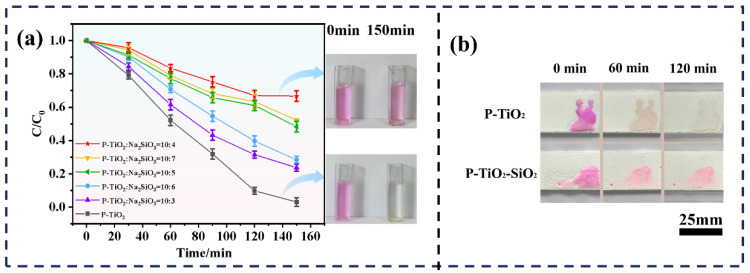
Degradation of RhB by the P-TiO_2_ and P-TiO_2_-SiO_2_ coatings: (**a**) degradation rate, (**b**) degradation effect.

The photocatalytic performances of the P-TiO_2_ and P-TiO_2_-SiO_2_ samples prepared with different mass ratios of P-TiO_2_ to Na_2_SiO_3_ were investigated. Figure 10a shows the degradation of RhB by these samples. P-TiO_2_ illustrated the best photocatalytic activity, and 96.93% of RhB was degraded in 150 min. In contrast, the photocatalytic property of P-TiO_2_-SiO_2_ was lower, and P-TiO_2_-SiO_2_ (P-TiO_2_:Na_2_SiO_3_ = 10:4) showed the lowest degradation rate of 33.33%. This indicated that the SiO_2_ coating effectively reduced the photocatalytic activity of P-TiO_2_. It was due to the fact that the active sites on the P-TiO_2_ surface was covered by SiO_2_. Additionally, the SiO_2_ coating might hinder the effective separation of photogenerated electrons and holes. It reduced its photocatalytic activity. These results demonstrated that the photocatalytic performance of P-TiO_2_ can be reduced by the SiO_2_ coating.

To observe intuitively the photocatalytic property of P-TiO_2_ and P-TiO_2_-SiO_2_ on organic compounds, the coatings were prepared by spraying P-TiO_2_ and P-TiO_2_-SiO_2_ onto glass slides. Then, the coatings were used to degrade the RhB solution (50 ppm) under UV irradiation, as shown in Figure 10b. After UV irradiation for 60 min, the color of RhB on the P-TiO_2_ coating surface faded noticeably. RhB was almost completely decolorized in 120 min. In the same conditions, the color of RhB on the P-TiO_2_-SiO_2_ coating surface did not noticeably fade. This further confirmed that the SiO_2_ coating effectively reduced the photocatalytic activity of P-TiO_2_ and enhanced its UV-aging resistance.

#### 3.2.3. Photoelectrochemical Performance of P-TiO_2_-SiO_2_

**Figure 11:**
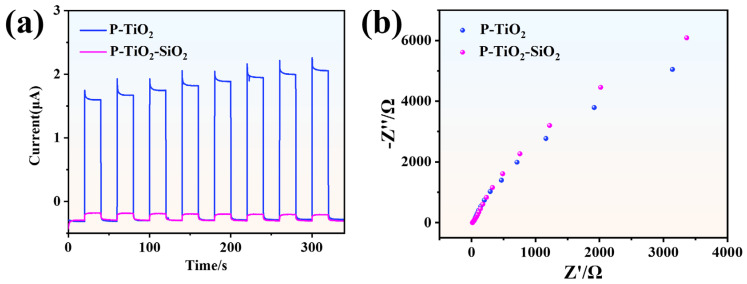
(**a**) Photocurrent and (**b**) impedance of P-TiO_2_-SiO_2_ and P-TiO_2_.

Figure 11a shows the transient photocurrent of P-TiO_2_ and P-TiO_2_-SiO_2_. It can be observed from the intensity of the transient photocurrent that P-TiO_2_-SiO_2_ hardly generated any photocurrent. On the contrary, the photocurrent density of P-TiO_2_ was significantly higher than that of P-TiO_2_-SiO_2_. It was attributed to the SiO_2_ coating, which scattered and reflected most of the UV light. It inhibited the separation and migration of photogenerated charges. As a result, the photoactivity and photocatalytic performance of P-TiO_2_ were decreased.

Electrochemical impedance spectroscopy (EIS) was used to study the charge transfer efficiency of P-TiO_2_ and P-TiO_2_-SiO_2_. The arc radius represented the electrochemical impedance of the samples. The P-TiO_2_-SiO_2_ coating exhibited a larger arc radius, as shown in Figure 11b, indicating that the SiO_2_ coating reduced the transfer rate of photogenerated carriers. This further confirmed that the photocatalytic performance of P-TiO_2_ was weakened after being coated with SiO_2_.

### 3.3. Structural Basis of the P-TiO_2_-SiO_2_/HDTMS Coating

The structural foundation of the P-TiO_2_-SiO_2_/HDTMS coating was the micro–nano binary structure formed by loading amorphous nano-SiO_2_ onto the P-TiO_2_ surface. The morphology and structure of P-TiO_2_-SiO_2_ were characterized by SEM, TEM, XRD, and XRF.

#### 3.3.1. Morphology of P-TiO_2_-SiO_2_

**Figure 12:**
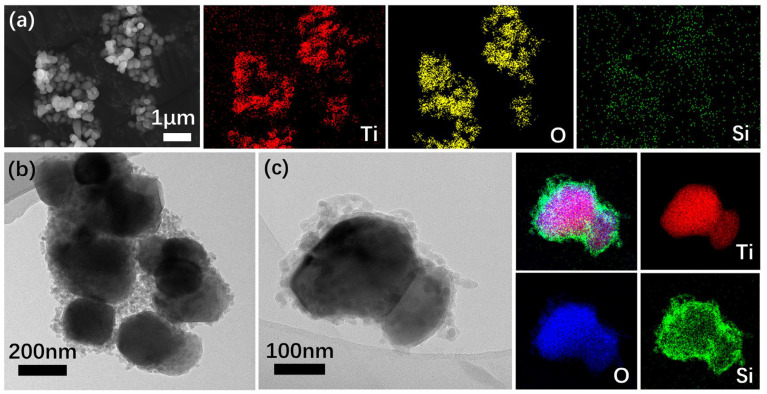
(**a**) SEM and elemental distribution of P-TiO_2_-SiO_2_; (**b**,**c**) TEM and elemental distribution of P-TiO_2_-SiO_2_.

Figure 12a shows the SEM image of P-TiO_2_-SiO_2_ composite particles. A small amount of agglomeration can be observed in the P-TiO_2_ particles, which was attributed to its small particle size, high surface energy, and absorption of moisture during sampling. Figure 12b,c present the TEM images of the P-TiO_2_-SiO_2_ coating. The P-TiO_2_ surface was uniformly coated with SiO_2_ particles, and the particle size of the P-TiO_2_-SiO_2_ composite was approximately 200 nm. The average thickness of the SiO_2_ coating layer was about 20–30 nm. The elemental distribution of Si, O, and Ti in P-TiO_2_-SiO_2_ revealed that the positions of the element Si were consisted with the contours of the element Ti, and it was uniformly distributed. It demonstrated that the nanoscale particles uniformly loaded on the surface of P-TiO_2_ were SiO_2_.

#### 3.3.2. Phase Component of P-TiO_2_-SiO_2_

**Figure 13:**
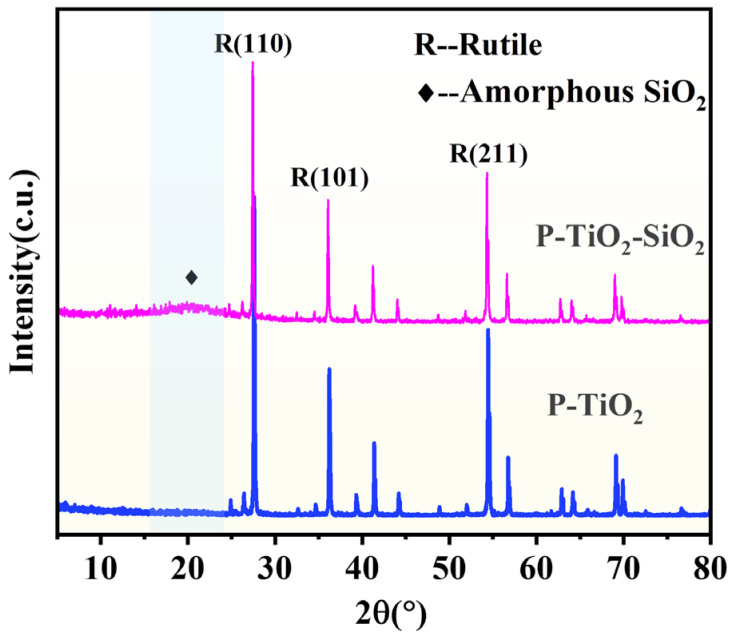
XRD patterns of P-TiO_2_ and P-TiO_2_-SiO_2_.

Figure 13 shows the XRD results of P-TiO_2_ and P-TiO_2_-SiO_2_. It illustrated that P-TiO_2_ was rutile. A characteristic peak of the amorphous material appeared at around 2θ = 21° in a P-TiO_2_-SiO_2_ pattern, and the intensity of the rutile diffraction peaks decreased slightly. Based on the reaction process and mechanism, it was inferred that SiO_2_ had been coated on the surface of P-TiO_2_ successfully.

#### 3.3.3. Chemical Composition of P-TiO_2_-SiO_2_

Table 1 presents the XRF results of P-TiO_2_ and P-TiO_2_-SiO_2_. In P-TiO_2_, the content of TiO_2_ was approximately 99.50%, indicating high purity of the raw material. Meanwhile, the content of SiO_2_ was only 0.01%, which might be the impurity in P-TiO_2_. In P-TiO_2_-SiO_2_, the content of SiO_2_ increased to 12.54%, and the content of TiO_2_ decreased to 86.59%. This confirmed that SiO_2_ had been loaded onto the surface of P-TiO_2_ successfully, and its quantity was 12.53%.

### 3.4. Combination of Raw Materials

The combination of raw materials directly affects the stability of the coating in practical applications. The combination of raw materials was analyzed by FT-IR and XPS.

#### 3.4.1. FT-IR Analysis

**Figure 14:**
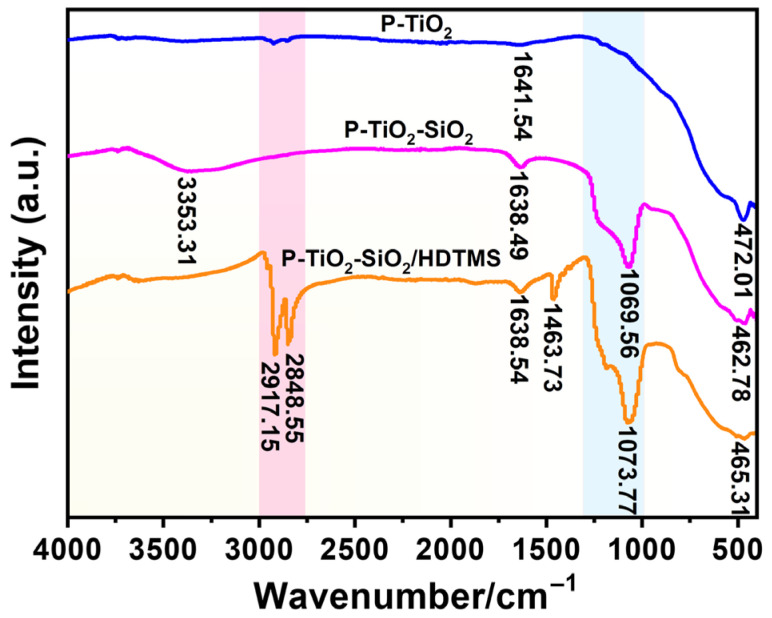
FT-IR spectra of P-TiO_2_, P-TiO_2_-SiO_2_, and P-TiO_2_-SiO_2_/HDTMS.

Figure 14 shows the FT-IR spectra of P-TiO_2_, P-TiO_2_-SiO_2_, and P-TiO_2_-SiO_2_/HDTMS. The peak at 472.01 cm^−1^ in the P-TiO_2_ spectrum was the peak of a Ti-O-Ti bond. A new absorption peak appeared at 1069.56 cm^−1^ in the spectrum of the P-TiO_2_-SiO_2_ composite particles, which can be attributed to the asymmetric stretching vibration of Si-O bonds in amorphous hydrated SiO_2_. This proved that SiO_2_ had been successfully coated on the surface of P-TiO_2_. The broad peak at 3353.31 cm^−1^ can be ascribed to the -OH anti-symmetric stretching vibration peak of structured water, and the peak near 1638.49 cm^−1^ can be ascribed to the H-O-H bending vibration peak of water [49]. Its relative strength increased, indicating that the formation of the SiO_2_ coating layer increased the hydroxyl content on the surface of P-TiO_2_ particles. The peaks of the H-O-H bending vibration (1638.49 cm^−1^) and Ti-O-Ti bond (462.78 cm^−1^) can be observed in the P-TiO_2_-SiO_2_ spectrum. They shifted obviously compared with the corresponding characteristic peaks in the P-TiO_2_ spectrum. It was inferred that SiO_2_ and P-TiO_2_ formed Si-O-Ti bonds. Therefore, it was believed that SiO_2_ and P-TiO_2_ formed a strong bond through chemical reactions. In the FT-IR spectrum of P-TiO_2_-SiO_2_/HDTMS, the vibrational absorption peaks of the -CH_3_ and -CH_2_- groups can be observed at 2917.15 cm^−1^ and 2848.55 cm^−1^, and the peak at 1463.73 cm^−1^ was the bending vibration of the C-H bond. This indicated that P-TiO_2_-SiO_2_ had been successfully modified by HDTMS. In addition, the position of the peak of the Ti-O-Ti bond (465.31 cm^−1^) and the asymmetric stretching vibration of the Si-O bond (1073.77 cm^−1^) had significantly shifted compared to the P-TiO_2_-SiO_2_ spectrum. It can be inferred that a chemical bond had formed between HDTMS and P-TiO_2_-SiO_2_ [50,51].

#### 3.4.2. XPS Analysis

**Figure 15:**
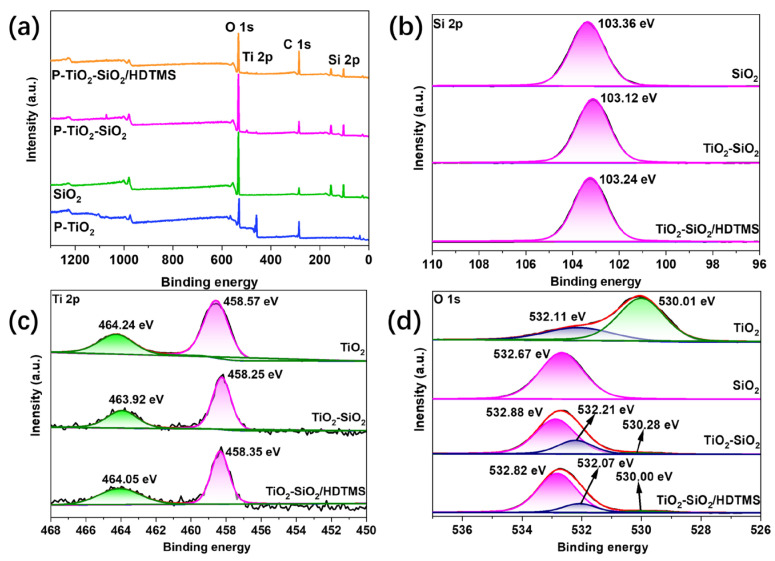
(**a**) XPS spectra of P-TiO_2_, SiO_2_, P-TiO_2_-SiO_2_, and P-TiO_2_-SiO_2_/HDTMS samples; (**b**) Si 2; (**c**) Ti 2p; and (**d**) O 1s.

Figure 15 shows the XPS results of P-TiO_2_, SiO_2_, P-TiO_2_-SiO_2_, and P-TiO_2_-SiO_2_/HDTMS. Figure 15a shows the full-spectrum scan image. In the spectrum of P-TiO_2_-SiO_2_, the peaks of the elements Si, Ti, and O can be seen. And the peak of the Ti element in the P-TiO_2_-SiO_2_ spectrum was significantly reduced compared to the peak of the Ti element in the P-TiO_2_ spectrum. This confirmed the successful loading of SiO_2_ on the P-TiO_2_ surface. The peak of the C element in the spectrum of P-TiO_2_-SiO_2_/HDTMS was significantly enhanced. This illustrated that P-TiO_2_-SiO_2_ had been successfully modified by HDTMS. Figure 15b–d show high-resolution scanning images of the samples. The peaks of Si 2p located around 103.3 eV can be seen clearly in the high-resolution spectra of SiO_2_ and P-TiO_2_-SiO_2_, as shown in Figure 15b. It indicated the presence of the SiO_2_ phase. In Figure 15b,c, the binding energy of Ti 2p and Si 2p on the surface of P-TiO_2_ coated with SiO_2_ shifted significantly. The peak located at 532.88 eV in the O 1s high-resolution spectrum of P-TiO_2_-SiO_2_ in Figure 15d can be attributed to the Si-O bond of SiO_2_. It shifted significantly compared to the peak position (532.67 eV) in the SiO_2_ spectrum. The peak located at 530.28 eV belonged to the Ti-O bond, which shifted compared to that in P-TiO_2_. This may be due to the changes in the chemical environment of Si and Ti. In addition, the hydroxyl characteristic peak located at 532.07 eV in the P-TiO_2_-SiO_2_/HDTMS spectrum shifted clearly and weakened in intensity compared to that in P-TiO_2_-SiO_2_. A chemical bond may be formed between HDTMS and P-TiO_2_-SiO_2_.

### 3.5. Properties of the Samples

The P-TiO_2_-SiO_2_ composite particles, the P-TiO_2_-SiO_2_/HDTMS coating, and the P-TiO_2_-SiO_2_/HDTMS coating with epoxy resin were investigated in this study. In order to clearly distinguish the properties and relationships of the three samples mentioned above, their properties are listed in Table 2. It can be seen from the table that the P-TiO_2_-SiO_2_ composite particles had UV-aging resistance. They were the raw materials for preparing the P-TiO_2_-SiO_2_/HDTMS coatings. The P-TiO_2_-SiO_2_/HDTMS coating was obtained by modifying the P-TiO_2_-SiO_2_ composite particles with HDTMS and spraying. The P-TiO_2_-SiO_2_/HDTMS coating showed UV-aging resistance and superhydrophobic properties. The mechanical strength of the P-TiO_2_-SiO_2_/HDTMS coating can be improved effectively by adding epoxy resin during the preparation of the coating. Therefore, the P-TiO_2_-SiO_2_/HDTMS coating with epoxy resin showed UV-aging resistance, superhydrophobicity, and mechanical strength.

## 4. Conclusions

In summary, the amorphous nano-SiO_2_ was coated on the surface of P-TiO_2_ using the acid precipitation method to prepare P-TiO_2_-SiO_2_ composite particles. The superhydrophobic P-TiO_2_-SiO_2_/HDTMS self-cleaning coating with UV-aging resistance was prepared by modifying and spraying. The WCA and WSA of the P-TiO_2_-SiO_2_/HDTMS coating surface were 154.9° and 1.3°, respectively, exhibiting superhydrophobic self-cleaning properties similar to the lotus leaf surfaces. This coating had excellent UV-aging resistance. After continuous irradiation in a UV accelerated aging chamber for 45 days, there was no significant change in the WCA and WSA of the P-TiO_2_-SiO_2_/HDTMS coating. The coating of SiO_2_ can effectively suppress the photocatalytic activity of P-TiO_2_ and significantly improve its UV-aging resistance. Therefore, the P-TiO_2_-SiO_2_/HDTMS coating maintained superhydrophobic self-cleaning performance for a long time.

## Figures and Tables

**Table 1:** XRF results of P-TiO_2_ and P-TiO_2_-SiO_2_.

Sample	TiO_2_ (%)	SiO_2_ (%)	K_2_O (%)	Al_2_O_3_ (%)	Na_2_O (%)	Others (%)
P-TiO_2_	99.50	0.01	0.16	0.08	0.01	0.24
P-TiO_2_-SiO_2_	86.59	12.54	0.03	0.11	0.52	0.21

**Table 2:** Properties of different samples.

	UV-Aging Resistance	Superhydrophobicity	Mechanical Strength
P-TiO_2_-SiO_2_ composite particles	√	×	×
P-TiO_2_-SiO_2_/HDTMS coating	√	√	×
P-TiO_2_-SiO_2_/HDTMS coating with epoxy resin	√	√	√

## Data Availability

Data are contained within the article and Supplementary Materials.

## Supplementary Materials

The following supporting information can be downloaded at https://www.mdpi.com/article/10.3390/nano15141127/s1, Figure S1. Effect of P-TiO_2_ and Na_2_SiO_3_ addition on the whiteness of P-TiO_2_-SiO_2_.
