# Supplementary material for: Preparation of Superhydrophobic P-TiO2-SiO2/HDTMS Self-Cleaning Coatings with UV-Aging Resistance by Acid Precipitation Method
Source: Nanomaterials (Basel). 2025 Jul 20;15(14):1127. doi: 10.3390/nano15141127 (PMC12298511; doi:10.3390/nano15141127)
Supplement: Supplementary file 1 [file nanomaterials-15-01127-s001.zip › nanomaterials-3756030-supplementary.pdf]

Preparation of Superhydrophobic $P\text{-}TiO_2\text{-}SiO_2$/HDTMS Self-Cleaning Coatings with UV-Aging Resistance by Acid Precipitation Method

Le Zhang [1], Ying Liu [2], Xuefeng Bai [1], Hao Ding [1,*], Xuan Wang [3], Daimei Chen [1,*] and Yihe Zhang [1]

1 Engineering Research Center of Ministry of Education for Geological Carbon Storage and Low Carbon Utilization of Resources, Beijing Key Laboratory of Materials Utilization of Nonmetallic Minerals and Solid Wastes, National Laboratory of Mineral Materials, School of Materials Science and Technology, China University of Geosciences, Xueyuan Road, Haidian District, Beijing 100083, China
2 Department of Mechanical, Materials and Manufacturing Engineering, The University of Nottingham, University Park, Nottingham NG7 2RD, UK
3 Laboratory and Equipment Management Department, China University of Geosciences, Xueyuan Road, Haidian District, Beijing 100083, China
* Correspondence: dinghao113@126.com or dinghao@cugb.edu.cn (H.D.); chendaimei@cugb.edu.cn (D.C.)

Experimental details

Measurement of water contact angles (WCAs) and water slide angles (WSAs)

The wettability of surfaces was characterized by measuring the WCAs and WSAs. Water droplets (8 μl) were used to measure the WCAs and WSAs, and they were determined by a contact angle meter (JC2000D, Shanghai Zhongchen Digital Technic Apparatus Co., Ltd., China). The WCAs and WSAs were determined by measuring each sample at five diverse positions.

Evaluation of UV-aging resistance

UV accelerated aging experiments were conducted using a Q8-UV1 UV accelerated weathering tester (Lab Companion Co., Ltd., Guangdong, China) with a test cycle of 24 h. The UV radiation intensity was 1000 mV/m^2, the distance from the light source (UVB-340 xenon lamp) to the sample was 254 mm, and the temperature during irradiation was maintained at 45°C. For sample preparation, $P\text{-}TiO_2\text{-}SiO_2$ composite particles prepared under different conditions were formulated into coatings. A 200 μm thick film was applied to glass plates using a wire rod coater, allowed to dry naturally, and then placed in the UV accelerated weathering tester for UV-aging experiments.

Evaluation of photocatalytic properties

The degradation performance of the samples was measured using a photochemical reactor (Phchem III, Beijing NBeT Technology Co., Ltd, China). 50 mg of samples and

50 mL of RhB solution (50 ppm) were mixed, and the photocatalytic degradation performance was estimated by (C/C_0) of RhB under UV irradiation by a 500 W high-pressure mercury lamp (365 nm) with the irradiance value of 200 mW/cm^2. The initial concentration of RhB was 10 ppm (C_0), and it was marked as C after degradation. The degradation rate can be calculated by $((C_0-C)/C_0*100\%)$.

Evaluation of photoelectrochemical performance

The photoelectrochemical performance of the samples was tested using a Zahner PP211 electrochemical workstation. A total of 10 mg of the powder was dispersed in 1 mL of an ultrapure water/ethanol mixed solution, followed by the addition of 50 μl of Nafion solution. The mixture was ultrasonicated for 30 min to form a uniform suspension, and 100 μl of the suspension was dropped onto an ITO electrode and dried at room temperature for photoelectrochemical testing. A three-electrode system (counter electrode: platinum electrode; reference electrode: Ag/AgCl/3M KCl; working electrode: ITO) was used for the tests. The electrolyte was a Na_2SO_4 solution (0.1 mol/L), and the light source was monochromatic light with a wavelength of 365 nm. The electrochemical methods used included current-time curve measurements and electrochemical impedance spectroscopy (EIS). The EIS tests were conducted under illumination, with a frequency range of 100 kHz to 0.1 Hz and an amplitude of 10 mV.

Characterizations

X-ray powder diffraction (XRD) was performed using a D8 Advance instrument from Bruker AXS, Germany. The technical parameters were as follows: Cu Kα target (λ = 1.5418 Å), operating voltage of 40 kV, operating current of 40 mA, step size of 0.02°, scanning speed of 0.05 s/step, and a scanning angle range of 5° to 80°. Fourier transform infrared spectroscopy (FT-IR) of the samples was conducted using a SPEETRUM 100 Fourier transform infrared spectrometer from PERKIN ELMER (HK) LTD, Hong Kong, with a scanning range of 4000 cm^{-1} to 400 cm^{-1}. For sample preparation, KBr was used as the medium, with a sample-to-KBr mass ratio of 1:50. Scanning electron microscopy (SEM) was performed using a Hitachi SU-8010 cold field emission scanning electron microscope from Hitachi High-Technologies, Japan. Transmission electron microscopy (TEM) was carried out using a JEM-2100F transmission electron microscope from Japan, equipped with an XFlash 5030T X-ray energy dispersive spectrometer from Bruker, Germany. X-ray photoelectron spectroscopy (XPS) was conducted using a Thermo Escalab 250Xi X-ray photoelectron

spectrometer from Thermo Fisher Scientific, USA, with Al Kα radiation, an operating voltage of 200 W, and data calibrated using C 1s (248.8 eV). Peak deconvolution was performed using XPS Peak software. X-ray fluorescence spectroscopy (XRF) was performed using an ARL AdvantX Intellipower™ 3600 X-ray fluorescence spectrometer from Thermo Fisher Scientific, USA. The whiteness of the powder samples was measured using a WSB-2 whiteness meter from Shanghai Xinrui Instrument Co., Ltd., with high-purity $BaSO_4$ as the reference (whiteness set to 100%). Chromaticity values (L*, a*, b*) were measured using an SP-60X-Rite (USA) color difference meter. The formulas for calculating the yellowing index (Δb*) and color difference (ΔE*) are as follows: Δb* (yellowing index) = b_I - b_0; ΔE* (color difference)= $\sqrt{(L_n^* - L_0^*)^2 + (a_n^* - a_0^*)^2 + (b_n^* - b_0^*)^2}$, In the formula, L_0^*, a_0^*, b_0^*andL_n^*, a_n^*, b_n^*represent the colorimetric values of the coating before and after irradiation, respectively, indicating the three attributes of color: lightness, red-green, and yellow-blue.

Whiteness

The influence of the ratio of P-TiO_2 to Na_2SiO_3 on the whiteness of P-TiO_2-SiO_2 composite powder was investigated. The whiteness of P-TiO_2 and P-TiO_2-SiO_2 is shown in Fig. 5. The whiteness of P-TiO_2 was 91.0%, and the whiteness of P-TiO_2-SiO_2 was above 92.3%. Specifically, when the mass ratio of P-TiO_2 and Na_2SiO_3 was 10:4, the whiteness was 93.4%. It increased by 2.4% compared to P-TiO_2. This was because the whiteness of pigments largely depended on their ability to reflect and scatter light. The coating of SiO_2 can effectively enhance the reflection and scattering forces of P-TiO_2. Therefore, the whiteness of P-TiO_2-SiO_2 was significantly higher than that of P-TiO_2. The improvement of whiteness was of great significance for the practical application of coatings.

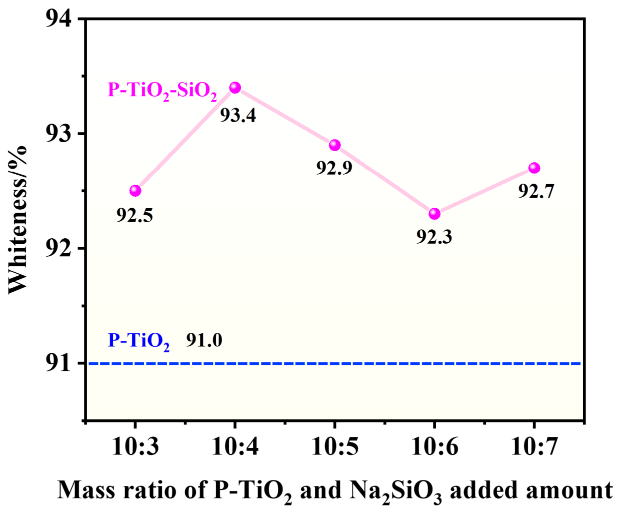

Figure S1. Effect of P-TiO_2 and Na_2SiO_3 addition on the whiteness of P-TiO_2-SiO_2.
